# Septins promote caspase activity and coordinate mitochondrial apoptosis

**DOI:** 10.1002/cm.21696

**Published:** 2022-05-09

**Authors:** Hoan Van Ngo, Stevens Robertin, Dominik Brokatzky, Magdalena K. Bielecka, Damián Lobato‐Márquez, Vincenzo Torraca, Serge Mostowy

**Affiliations:** ^1^ Department of Infection Biology London School of Hygiene and Tropical Medicine London UK; ^2^ School of Life Sciences University of Westminster London UK

**Keywords:** apoptosis, cytoskeleton, mitochondria, septins, *Shigella*, zebrafish

## Abstract

Apoptosis is a form of regulated cell death essential for tissue homeostasis and embryonic development. Apoptosis also plays a key role during bacterial infection, yet some intracellular bacterial pathogens (such as *Shigella flexneri*, whose lipopolysaccharide can block apoptosis) can manipulate cell death programs as an important survival strategy. Septins are a component of the cytoskeleton essential for mitochondrial dynamics and host defense, however, the role of septins in regulated cell death is mostly unknown. Here, we discover that septins promote mitochondrial (i.e., intrinsic) apoptosis in response to treatment with staurosporine (a pan‐kinase inhibitor) or etoposide (a DNA topoisomerase inhibitor). Consistent with a role for septins in mitochondrial dynamics, septins promote the release of mitochondrial protein cytochrome c in apoptotic cells and are required for the proteolytic activation of caspase‐3, caspase‐7, and caspase‐9 (core components of the apoptotic machinery). Apoptosis of HeLa cells induced in response to infection by *S. flexneri* Δ*galU* (a lipopolysaccharide mutant unable to block apoptosis) is also septin‐dependent*.* In vivo, zebrafish larvae are significantly more susceptible to infection with *S. flexneri* Δ*galU* (as compared to infection with wildtype *S. flexneri*), yet septin deficient larvae are equally susceptible to infection with *S. flexneri* Δ*galU* and wildtype *S. flexneri*. These data provide a new molecular framework to understand the complexity of mitochondrial apoptosis and its ability to combat bacterial infection.

## 1 INTRODUCTION

Apoptosis, a non‐inflammatory form of regulated cell death used to eliminate damaged cells, is essential for tissue homeostasis and embryonic development (Danial & Korsmeyer, 2004). The core apoptotic machinery involves caspases (Thornberry & Lazebnik, 1998) that reside in the cytosol as zymogens/proenzymes and consist of two major subgroups: initiator caspases (caspase‐8, caspase‐9) and effector/executioner caspases (caspase‐3, caspase‐7) (Salvesen & Ashkenazi, 2011). Apoptotic stimuli induce activation of initiator caspases through dedicated signaling pathways, initiated intrinsically (i.e., mitochondria‐mediated pathway) or extrinsically (i.e., receptor‐mediated pathway) (Ashkenazi & Dixit, 1998; Bratton & Salvesen, 2010). Following activation, initiator caspases trigger proteolytic stimulation of effector caspase‐3 and caspase‐7 zymogens that enable the execution phase of the apoptotic cell death program by cleaving functionally important proteins within a cell (Ashkenazi & Salvesen, 2014).

Septins are a poorly understood component of the cytoskeleton discovered from their role in cytokinesis (Mostowy & Cossart, 2012; Spiliotis & Nakos, 2021; Woods & Gladfelter, 2021). In humans, septins are organized into four groups based on amino acid sequence similarity: the SEPT2 group (SEPT1, SEPT2, SEPT4, SEPT5), SEPT3 group (SEPT3, SEPT9, SEPT12), SEPT6 group (SEPT6, SEPT8, SEPT10, SEPT11, SEPT14), and SEPT7 group (SEPT7). Members of each septin group assemble into hetero‐oligomeric complexes (including SEPT2‐SEPT6‐SEPT7‐SEPT9‐SEPT9‐SEPT7‐SEPT6‐SEPT2) and higher‐order structures (including filaments and rings). In the case of bacterial infection, septins are recognized for their role in host defense against the important human pathogen *Shigella flexneri* (Robertin & Mostowy, 2020; Van Ngo & Mostowy, 2019). Here, septins assemble into cage‐like structures that entrap dividing bacterial cells for targeting to autophagy (Krokowski et al., 2018; Lobato‐Márquez et al., 2021; Mostowy et al., 2010). Septins also interact with the cytosolic GTPase dynamin‐related protein 1 (Drp1) to enhance mitochondrial fission (Pagliuso et al., 2016; Sirianni et al., 2016). In the case of cell death, apoptosis‐related protein in the TGF‐β signaling pathway (ARTS), an unusual septin derived from alternative splicing of *SEPT4*, has been described to promote mitochondrial apoptosis (Larisch et al., 2000). Despite the evidence for ARTS in mitochondrial apoptosis (Edison et al., 2012; Edison et al., 2017; García‐Fernández et al., 2010; Gottfried, Rotem, Lotan, Steller, & Larisch, 2004; Larisch et al., 2000; Mamriev et al., 2020), a role for other septins in apoptosis was unknown.

Apoptosis is widely recognized as important for immune defense against bacterial invasion, yet pathogens deploy a variety of mechanisms to manipulate cell death pathways and promote infection (Naderer & Fulcher, 2018). In the case of *S. flexneri*, both inhibition and induction of apoptosis have been described. *S. flexneri* can inhibit apoptosis in epithelial cells by inhibiting the activation of caspase‐3 (Clark & Maurelli, 2007), and recent work identified that *S. flexneri* lipopolysaccharide (LPS) can directly bind to caspase‐3/‐7 via its O‐antigen moiety, blocking apoptosis signaling (Günther et al., 2020). On the other hand, work has shown that *S. flexneri* can induce apoptosis of infected cells via mitochondrial depolarization and caspase‐9 activation (Lembo‐Fazio et al., 2011). Taken together, the precise role of apoptosis in control of *S. flexneri* infection is complex and, in terms of host and pathogen survival, is the subject of great interest (Ashida, Suzuki, & Sasakawa, 2021).

In this study, we investigate a role for septins in mitochondrial apoptosis induced by pharmacological treatment and *S. flexneri* infection. We discover that septins (SEPT7 and SEPT2) are required for apoptotic cell death. Consistent with this, we show that septins promote the release of cytochrome c from mitochondria, as well as the activation of caspase‐3, caspase‐7, and caspase‐9. Finally, we highlight the role of septin‐mediated apoptosis during *S. flexneri* infection in vitro using human epithelial cells and in vivo using zebrafish larvae.

## 2 RESULTS

### 2.1 Septins are required for apoptotic cell death

**FIGURE 1:**
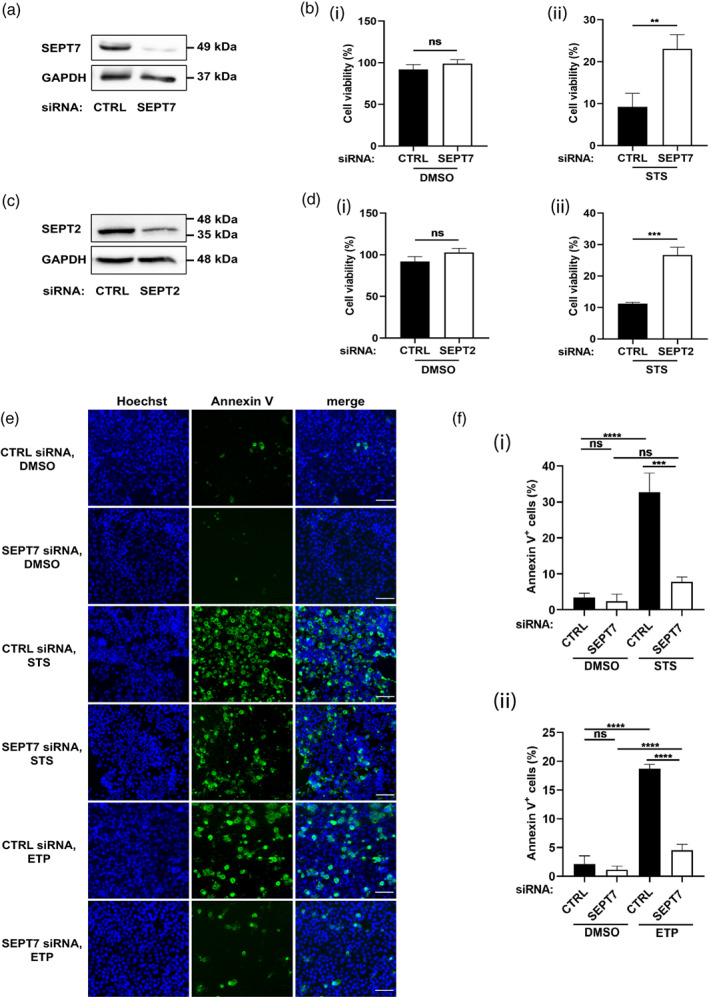
Septin‐depleted cells undergo significantly less apoptosis. (a) HeLa cells were transfected for 72 hr with control (CTRL) siRNA or a siRNA targeting SEPT7. Effect of siRNA on SEPT7 expression. A representative Western blot of HeLa cell lysates is shown. GAPDH was used as a loading control. (b) Impact of siRNA targeting SEPT7 on viability of HeLa cells in (i) untreated or (ii) STS‐treated conditions. Cell viability is measured using MTT assays as described in Materials and Methods. Data are mean ± SEM from three independent experiments. **p = .0069 by two‐tailed Student's *t* test. (c) HeLa cells were transfected for 72 hr with control (CTRL) siRNA or a siRNA targeting SEPT2. Effect of siRNA on SEPT2 expression. A representative Western blot of HeLa cell lysates is shown. (d) Impact of siRNA targeting SEPT2 on viability of HeLa cells in (i) untreated or (ii) STS‐treated conditions. Cell viability is measured using MTT assays as described in Materials and Methods. Data are mean ± SEM from three independent experiments. ***p = .0004 by two‐tailed Student's *t* test. (e) Control and SEPT7‐depleted cells were treated with DMSO or 1 μM STS or 500 μM ETP for 10 hr and labeled with Alexa488‐Annexin V (Annexin V) and Hoechst. Representative widefield microscopy images showing Annexin V in green and Hoechst in blue. Scale bar = 100 μm. (f) The % of Annexin V positive cells is calculated in Fiji by dividing the number of cells that displayed Annexin V labeling by the total number of cells identified by nuclear Hoechst labeling, then multiplying by 100. Each bar represents the mean % ± SEM from three independent experiments (a minimum of 1,000 cells were counted per condition separated in three independent experiments). ****p < .0001by two‐way ANOVA.

To test a role for septins in apoptosis of human epithelial cells (HeLa), small interfering RNA (siRNA) was used to deplete SEPT7, and staurosporine (STS, a pan‐kinase inhibitor) was used to induce mitochondrial apoptosis (Figure 1a). We observed that 24 hr incubation of 1 μM STS dramatically reduced the viability of control cells (9.2 ± 1.9% cells survived), but only partially reduced the viability of SEPT7‐depleted cells (23.1 ± 2.0% cells survived) (Figure 1b). Similar results are observed for SEPT2‐depleted cells (Figure 1c, d). To visualize apoptosis in STS‐treated cells, samples were fluorescently labeled with Annexin V (to identify phosphatidylserine externalization, a hallmark of apoptotic cells) and Hoechst (to visualize nuclei) (Figure 1e). In agreement with cell viability results, the proportion of Annexin V‐positive cells is significantly reduced (4.2 ± 0.1 fold) in SEPT7‐depleted cells, as compared to control cells (Figure 1f).

We next used etoposide, a drug that inhibits topoisomerase II causing errors in DNA replication, to induce mitochondrial apoptosis. Consistent with results obtained for STS‐treated cells, depletion of SEPT7 significantly reduced (4.2 ± 0.8 fold) the proportion of Annexin V‐positive cells, as compared to control cells (Figure 1e, f). Together, these results show that septins promote mitochondrial apoptosis of HeLa cells.

### 2.2 Septins are required for cytochrome c release

**FIGURE 2:**
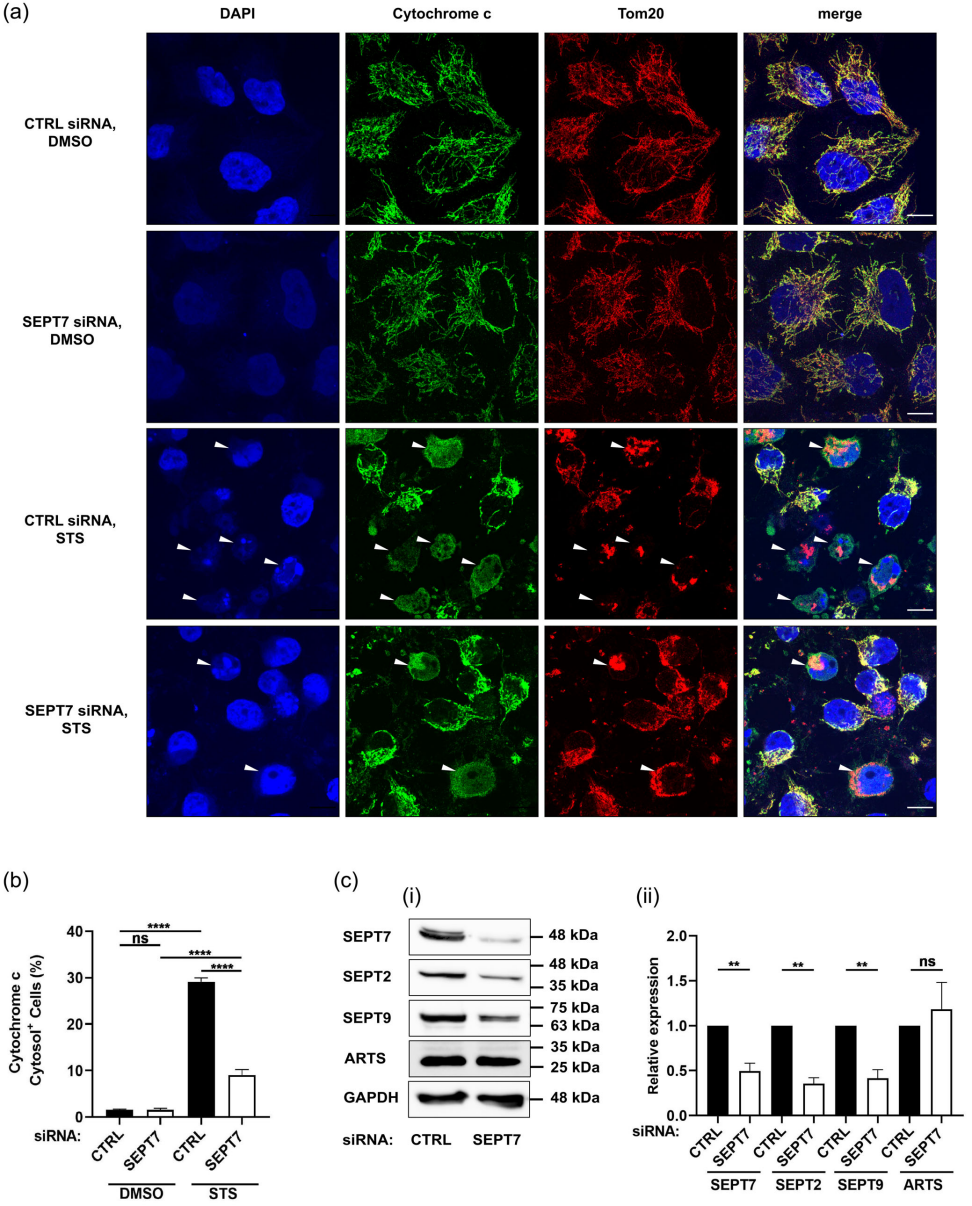
Cytochrome c release is significantly reduced in SEPT7‐depleted cells. HeLa cells were transfected with control or SEPT7 siRNA, treated with DMSO or 1 μM STS for 10 hr before being fixed and labeled with an anti‐cytochrome c and anti‐Tom20 antibodies and DAPI. (a) Representative confocal microscopy images showing cytochrome c in green, Tom20 in red and DAPI in blue. Arrowheads indicate cells releasing cytochrome c. Scale bar = 10 μm. (b) The percentage of cells releasing cytochrome c is calculated in ImageJ by counting cells that exhibited cytosolic (rather than mitochondrial) cytochrome c labeling, and dividing by the total number of cells, then multiplying by 100. Each bar represents the mean % ± SEM from three independent experiments (a minimum of 1,000 cells were counted per condition separated in three independent experiments). ****p < .0001 by two‐way ANOVA. (c) SEPT7 and ARTS act in different pathways. (i) HeLa cells were treated with control or SEPT7 siRNA for 72 hr. Whole‐cell lysates were immunoblotted for SEPT7, SEPT2, SEPT9, and ARTS. GAPDH was used as a loading control. Data are representative Western blots of three independent experiments. (ii) Densitometry analysis of the bands was performed using ImageJ. Data are mean ± *SEM* from three independent experiments. **p < .01 by two‐tailed Student's *t* test

Cytochrome c is located in the mitochondrial intermembrane space under normal conditions, but under apoptotic conditions is released to the cytosol for the activation of initiator caspases (Bock & Tait, 2020; Dorstyn, Akey, & Kumar, 2018). Considering that septins are well known to play a key role in mitochondrial dynamics (Pagliuso et al., 2016; Sirianni et al., 2016), we hypothesized that septins can also promote the release of cytochrome c when mitochondrial apoptosis is stimulated. We treated control and SEPT7‐depleted cells with STS and examined cytochrome c release using confocal microscopy and immunostaining of cytochrome c (as well as Tom20, a mitochondrial marker) (Figure 2a). In this case, the number of SEPT7‐depleted cells releasing cytochrome c is significantly reduced (3.2 ± 0.4 fold), as compared to control cells (Figure 2b).

Previous work has shown that ARTS, a variant of SEPT4, promotes mitochondrial apoptosis (Larisch et al., 2000). To investigate if SEPT7 depletion compromises the activation of apoptosis via ARTS, we quantified protein levels of ARTS and other septins in SEPT7‐depleted conditions. Consistent with previous work, we showed that the depletion of SEPT7 significantly reduced SEPT2, SEPT7, and SEPT9 protein levels (Estey et al., 2010; Lobato‐Marquez et al., 2019), but ARTS protein levels are not affected by SEPT7 depletion (Figure 2c). These results indicate that the requirement of SEPT7 in mitochondrial apoptosis is not due to the regulation of ARTS.

### 2.3 Septins are required for activation of caspase‐3/ ‐7/, and ‐9

**FIGURE 3:**
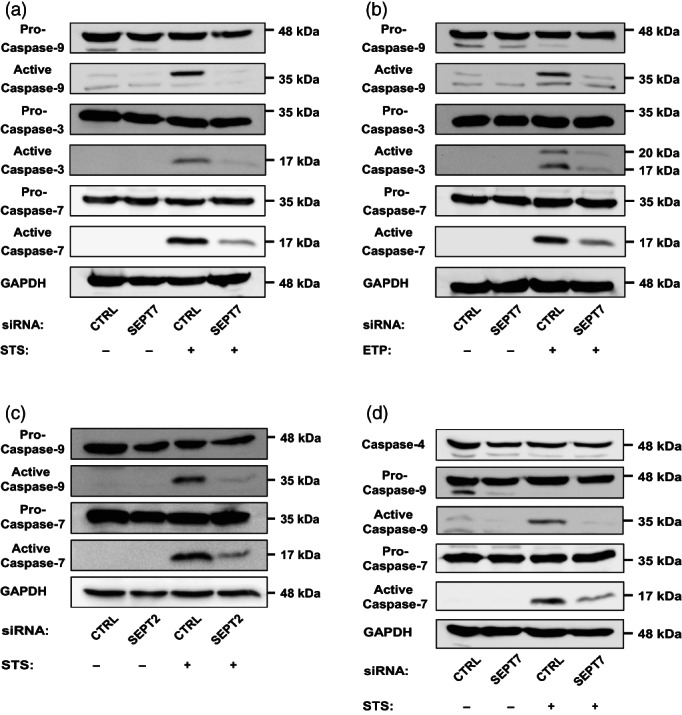
Initiator and executioner caspase cleavage is significantly reduced when septins are depleted. (a), (b) HeLa cells were transfected with control or SEPT7 siRNA for 72 hr. Cells were then treated with DMSO or (a) 1 μM STS or (b) 500 μM ETP for 10 hr. Whole‐cell lysates were immunoblotted against caspase‐3, cleaved caspase‐3, caspase‐7, caspase‐9, and GAPDH (used as loading control). Data are representative of three independent experiments. (c) SEPT2 is required for caspase activation. HeLa cells were transfected with control or SEPT2 siRNA for 72 hr. Cells were then treated with DMSO or 1 μM STS for 10 hr. Whole‐cell lysates were immunoblotted with antibodies to caspase‐7, caspase‐9, and GAPDH. Data are representative of three independent experiments. (d) HeLa cells were transfected with control or SEPT7 siRNA for 72 hr. Cells were then treated with DMSO or 1 μM STS for 10 hr. Whole‐cell lysates were immunoblotted with antibodies to caspase‐4, caspase‐7, caspase‐9, and GAPDH. Data are representative of three independent experiments

Activation of effector caspases (caspase‐3 and caspase‐7) occurs in two steps, beginning with initial cleavage by active caspase‐9 to form the p20 subunit. After this cleavage, caspase‐3 or caspase‐7 removes its own prodomain to generate the active p17 subunit. To understand how septins promote mitochondrial apoptosis, we measured the effect of septin depletion on STS‐ or etoposide‐induced activation of caspase‐9 by Western blotting (Figure 3a, b). In both cases, SEPT7 depletion caused a significant decrease in levels of active caspase‐9, as compared to control cells. Consistent with a role for septins in mitochondrial apoptosis, SEPT7 depletion caused a significant decrease in levels of active caspase‐3 and caspase‐7, as compared to control cells (Figure 3a, b). Similar results are observed for SEPT2‐depleted cells (Figure 3c). Importantly, SEPT7 depletion had no significant effect on levels of caspase‐4, an inflammatory caspase with no role in STS‐mediated mitochondrial apoptosis (Figure 3d).

**FIGURE 4:**
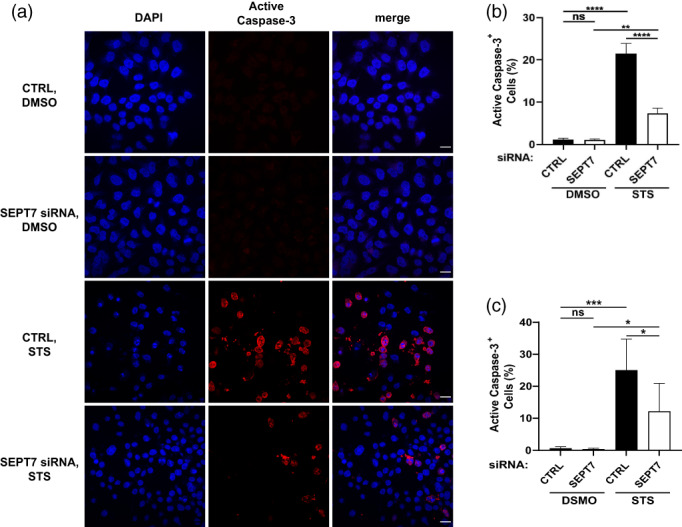
SEPT7 is required for the activation of caspase‐3. (a) Control and SEPT7‐depleted cells were treated with DMSO or STS, and stained with cleaved anti‐caspase‐3 antibody and DAPI. Representative confocal microscopy images showing cleaved caspase‐3 in red and DAPI in blue. Scale bar = 100 μm. (b) The percentage of cleaved caspase‐3‐stained cells was calculated in Fiji by dividing the number of cells that displayed cleaved caspase‐3 staining by the total number of cells, then multiplying by 100. Each bar represents the mean % ± SEM from three independent experiments (a minimum of 1,000 cells were counted per condition separated in three independent experiments). **p < .01, ****p < .0001 by two‐way ANOVA. (c) HeLa cells (CTRL or SEPT7 siRNA transfected) were treated with 1 μM staurosporine for 5 hr, followed by staining of active caspase‐3 and analyzed by flow cytometry (a minimum of cells were counted per condition separated in three independent experiments). *p < 0.05, ***p < 0.001 by two‐way ANOVA

Next, we imaged HeLa cells labeled for active caspase‐3 using confocal microscopy (Figure 4a). Consistent with results obtained by Western blot, SEPT7‐depleted cells showed a significant decrease (3.0 ± 0.6 fold) in the number of apoptotic cells labeled for active caspase‐3, as compared to control cells (Figure 4b). Similar results are obtained when we labeled for active caspase‐3 and performed flow cytometry analysis on siRNA‐treated cells (Figure 4c). Together, these results highlight an important role for septins in the activation of caspase‐3/ ‐7/, and ‐9 during mitochondrial apoptosis.

### 2.4 Testing the role of septin‐mediated apoptosis during *S. flexneri* infection

**FIGURE 5:**
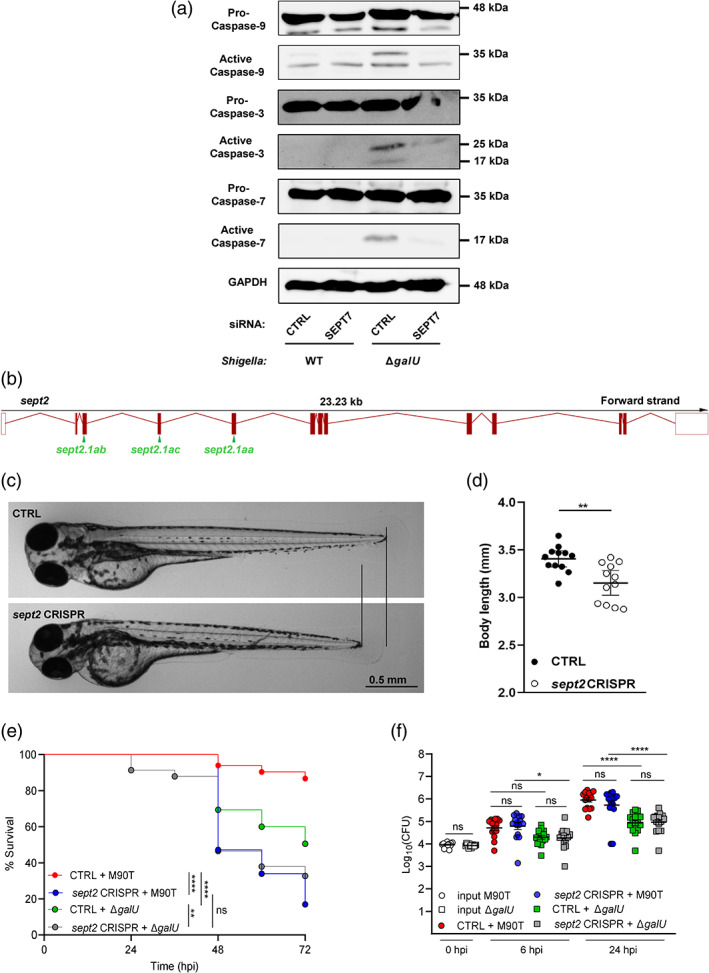
Septins are required for apoptosis induced by *Shigella* infection. (a) HeLa cells were transfected with control or SEPT7 siRNA for 72 hr. Cells were then infected with *S. flexneri* M90T or the *S. flexneri* Δ*galU* mutant for 4 hr. Whole‐cell lysates were immunoblotted against caspase‐3, caspase‐7, and caspase‐9. GAPDH was used as a loading control. Data are representative of three independent experiments. (b) Schematic representation of CRISPR/Cas9 targeting of *sept2*. Three crisprRNA (crRNA, green arrows) were designed, targeting three consecutive exons (red boxes) of the zebrafish *sept2* gene. (c) Representative images of control (CTRL) and *sept2* crispants. Lines indicate a difference in overall body length between CTRL and *sept2* crispants. Images were acquired using Leica M205 FA stereomicroscope. (d) Quantification of difference in body length between CTRL and *sept2* crispants. Bars represent the mean ± SEM from three independent experiments (*n* = 12 larvae per bar). **p < .01 by Student's *t* test. (e) Survival assays of CTRL and *sept2* crispants injected with approximately 9,000 CFU of either *S. flexneri* WT or *S. flexneri* Δ*galU*. **p < .01, ****p < .0001 by log–rank (Mantel–Cox) test. Curves are cumulative of 3 independent experiments (*n* = 53–85 larvae per curve). Statistical tests for survival curves were performed using the log–rank (Mantel–Cox) test (which intrinsically take into consideration the overall trend of survival over the different times of observation), and are indicated for overall survival. (f) CFU enumeration of CTRL and *sept2* crispants injected with approximately 9,000 CFU of either *S. flexneri* WT or *S. flexneri* Δ*galU*. CFU were collected at 0, 6, and 24 hr post‐infection, and are indicated on the X‐axis. Bars represent the mean ± SEM from three independent experiments (*n* = 15 larvae per bar). *p < .05, ****p < .0001 by one‐way ANOVA. Statistical tests for CFU counts were performed using one‐way ANOVA, and are indicated for the individual timepoints

New work has shown that *S. flexneri* LPS can bind to caspase‐3/ ‐7 via its O‐antigen moiety, blocking apoptosis signaling (Günther et al., 2020). Considering this, we used a *S. flexneri* mutant lacking the O‐antigen and outer core components of LPS (Δ*galU*) (Lobato‐Márquez et al., 2021) to test for caspase activation during infection of HeLa cells. Consistent with a role for bacterial LPS in blocking apoptosis, *S. flexneri* Δ*galU* fails to inhibit the activation of caspase‐3/ ‐7/ and ‐9 as efficiently as wildtype *S. flexneri* (Figure 5a). Strikingly, the activation of caspase‐3/ ‐7/ ‐9 in response to *S. flexneri* Δ*galU* infection is dependent on SEPT7 (Figure 5a).

To investigate the role of septin‐mediated apoptosis during infection in vivo, we used the well‐established *S. flexneri*‐zebrafish infection model (Duggan & Mostowy, 2018; Gomes & Mostowy, 2020; Torraca & Mostowy, 2018). Zebrafish septins are highly homologous to human septins and all septin groups (SEPT2, SEPT3, SEPT6, SEPT7) are represented in zebrafish, though some septins are duplicated in zebrafish (Figure S1A). For example, three homologs of human *SEPT7* are present in zebrafish (*sept7a*, *sept7b*, *sept15*). In contrast, only one zebrafish *sept2* gene has been identified, and it has high homology to human *SEPT2* (Figure S1A). Moreover, RNAseq data from our lab (Torraca et al., 2019) suggested that *sept2* is the most expressed septin in zebrafish at the whole animal level (Figure S1B, Table S1). Considering this, to investigate the role of septin‐mediated apoptosis in vivo, we targeted zebrafish Sept2 using a highly effective CRISPR‐Cas9 method (Kroll et al., 2021) resulting in F0 knockout (Figure 5b, Figure S1C).

At 3 days post‐fertilization (dpf), *sept2* crispants displayed some growth impairment, as shown by a reduced body length (Figure 5c‐d); only larvae capable of normal hatching and free from major developmental abnormalities were selected for infection studies. *sept2* crispants are significantly more susceptible to wildtype *S. flexneri* infection as compared to control larvae (Figure 5e–f), highlighting an in vivo role for septins in host defense. Control larvae are significantly more susceptible to *S. flexneri* Δ*galU* as compared to control larvae infected with wildtype *S. flexneri*, but *sept2* crispants (i.e., where mitochondrial apoptosis is compromised) are not significantly more susceptible to *S. flexneri* Δ*galU* as compared to *sept2* crispants infected with wildtype *S. flexneri* (Figure 5e‐f).

## 3 CONCLUDING REMARKS

Investigations using cellular and animal models have shown that septins are crucial for sensing bacteria and for promoting effector mechanisms to eliminate them (Mostowy & Shenoy, 2015; Robertin & Mostowy, 2020; Van Ngo & Mostowy, 2019). Here, we discover that septins are required for mitochondrial apoptosis because they promote cytochrome c release and activation of caspase‐3, caspase‐7, and caspase‐9. We highlight the role of septin‐mediated apoptosis during *S. flexneri* infection in vitro using human epithelial cells and in vivo using zebrafish larvae. Further investigations into septin biology will help to decipher the molecular mechanisms underlying human diseases associated with mitochondrial apoptosis, including susceptibility to infection.

The similarity of results obtained with SEPT7 and SEPT2 strongly suggests that septin hetero‐oligomers (and not septin monomers or dimers) are required for their role in apoptosis. This is in contrast to the apoptotic role of ARTS which is viewed to act independently of other septins. ARTS is localized to the mitochondrial outer membrane (Edison et al., 2012). In response to apoptotic stimuli, ARTS translocates to the cytosol and directly binds and antagonizes anti‐apoptotic XIAP, leading to the release of non‐lethal active caspases (e.g., caspase 9) from XIAP. In turn, these caspases cleave substrates (e.g., Bid), resulting in mitochondrial outer membrane permeabilization (MOMP) and apoptosis. Work has shown that ARTS acts as a scaffold to bring XIAP into close proximity with anti‐apoptotic Bcl‐2 and promote its degradation (Edison et al., 2017). Considering work from our lab and others (Krokowski et al., 2018; Lobato‐Márquez et al., 2021; Mageswaran et al., 2021; Mostowy et al., 2010; Pagliuso et al., 2016; Sirianni et al., 2016) it is tempting to speculate that septins (including SEPT7 and SEPT2, but not ARTS) are recruited to mitochondrial constriction sites that present micron‐scale curvature. Here, septins may function as a scaffold to recruit Drp1 to mitochondrial fission sites (in healthy cells) or apoptotic foci (in apoptotic cells), and promote interactions of Drp1 with other proteins involved in MOMP (e.g., Bax). Consistent with this, new work has shown that Drp1 interacts with Bax at apoptotic foci resulting in MOMP and apoptosis (Jenner et al., 2022).

To test the role of septins in apoptosis in vivo, we took advantage of the *Shigella*‐zebrafish infection model (Duggan & Mostowy, 2018; Gomes & Mostowy, 2020; Torraca & Mostowy, 2018). We observe that, as compared to control larvae, *sept2* crispants are not more susceptible to *S. flexneri* Δ*galU* (i.e., bacteria that do induce apoptosis) as compared to wildtype *S. flexneri* (i.e., bacteria that do not induce apoptosis). In contrast, control larvae are more susceptible to *S. flexneri* Δ*galU* as compared to wildtype *S. flexneri*. Considering that survival of *sept2* crispants is not affected in this case, these data are in agreement with our hypothesis that septins are required for *S. flexneri* Δ*galU* to induce host cell death via apoptosis. However, given the pleiotropic role of septins in vivo, these results involving zebrafish infection are likely not solely because of Sept2's role in apoptosis. As compared to control larvae, *sept2* crispants are significantly more susceptible to wildtype *S. flexneri*. In agreement with previous work from our lab studying septins in zebrafish (Mazon‐Moya et al., 2017; Mostowy et al., 2013), these data suggest that the role of Sept2 in restricting bacterial infection may also be acting through septin cages, bacterial autophagy, and inflammation control. Relatively little is known about the role of apoptosis during *S. flexneri* infection in vivo. It will thus be of great interest to further study the link between septins and apoptosis using a zebrafish model of *Shigella* infection.

## 4 MATERIALS AND METHODS

### 4.1 Mammalian cell lines, bacterial strains, and media

The human epithelial cell line HeLa (ATCC CCL‐2) was grown at 37 °C and 5% CO_2_ in Dulbecco's modified Eagle medium (DMEM, #5796‐500ML, Sigma) supplemented with 10% fetal bovine serum (FBS, #10500‐064‐500ML, Gibco).

*S. flexneri* M90T (Mostowy et al., 2010) or *S. flexneri* Δ*galU* (lacking O‐antigen and outer core components of LPS) (Lobato‐Márquez et al., 2021) were grown in trypticase soy (TCS) agar containing 0.01% (w/v) congo red to select for red colonies, indicative of a functional type III secretion system (T3SS). TCS liquid cultures were inoculated with individual red colonies of *S. flexneri* M90T or *S. flexneri* Δ*galU* and were grown overnight at 37 °C with shaking. The following day, bacterial cultures were diluted in fresh prewarmed TCS (1:50 v/v) and cultured until an optical density (OD_600_) of 0.6.

### 4.2 siRNA transfections and chemical treatments

HeLa cells (7 × 10^4^) were plated in 6‐well plates (Thermo Scientific) for 24 hr and then transfected with selected siRNAs as previously described (Lobato‐Márquez et al., 2019; Mostowy et al., 2010; Sirianni et al., 2016). siRNA transfection was performed in DMEM with oligofectamine (Invitrogen) according to the manufacturer’s instructions. Cells were tested 72 hr after siRNA transfection. Control siRNA (ID#14614) and predesigned siRNA for SEPT2 (ID#14709 and ID#14614), SEPT4 (ID#142770), SEPT7 (ID#10323) or SEPT9 (ID#18228) are all from Ambion.

Cells were treated with 1 μM staurosporine (STS, #ALX‐380‐014‐C250, Enzo) or 500 μM etoposide (ETP, #E1383‐25MG, Sigma) for 10 hr. Equivalent volumes of Dimethyl sulfoxide (DMSO) were used as controls.

### 4.3 Measurement of cell viability

Viability of HeLa cells (Figures 1b and S1B) was measured by MTT (3‐[4,5‐deimethylthiazol‐2‐yl]‐2,5‐diphenyltetrazolium bromide) assays (Kumar, Nagarajan, & Uchil, 2018). After DMSO or STS treatments, cells were washed with pre‐warmed PBS and placed in serum‐free DMEM. MTT was added to each well to a final concentration of 1 mg/mL and plates were incubated for 45 min at 37 °C in 5% CO_2_. DMSO was added to each well (final volume 50%) and plates were agitated gently for 10 min at room temperature to dissolve the precipitate of the blue dyes. Samples were then diluted 1:1 with DMSO and measured the absorbance at the wavelength of 600 nm.

### 4.4 Apoptosis assays

For apoptosis assays (Figure 1c), approximately 2.5 × 10^4^ cells were seeded into Lab‐Tek 8‐well glass‐bottom chamber slides (#177445, Thermo Scientific) and transfected with siRNA 72 hr prior to DMSO, STS or ETP treatments. Alexa488‐Annexin V (#A13201, Invitrogen), and Hoechst (#62249, Thermo Scientific) were added directly to the media and incubated for 15 min at 37 °C in 5% CO_2_. All live imaging was performed at 37 °C in 5% CO_2_ and captured using a 10x lens on a Zeiss Axio Observer Z1 widefield epifluorescence microscope driven by ZEN Blue 2.3 software (Carl Zeiss).

### 4.5 Antibodies and Western blotting

Rabbit polyclonal antibodies used were anti‐caspase‐3 (ID#9662S, Cell Signaling), anti‐caspase‐9 (ID#9502S, Cell Signaling), anti‐cleaved caspase‐3 (ID#9661S, Cell Signaling), anti‐SEPT7 (ID#18991, IBL), anti‐SEPT9 (ID#A302‐353A) and anti‐Tom20 (ID#42406S, Cell Signaling). Mouse antibodies used were anti‐ARTS (ID#A4471‐1ML, Sigma), anti‐caspase‐4 (ID#M029‐3, MBL), anti‐caspase‐7 (ID#9494S, Cell Signaling), anti‐cytochrome c (ID#12963A, Cell Signaling), anti‐GAPDH (ID#ab8245, Abcam), and anti‐SEPT2 (ID#60075‐1‐Ig, Proteintech Europe). Secondary antibodies used were goat anti‐mouse (ID#P0260, Dako) or anti‐rabbit (ID#P0448, Dako) antibodies, both horseradish peroxidase‐conjugated. GAPDH was used as a loading control. All antibodies were diluted in blocking solution (Tris Buffered Saline containing 0.1% Tween 20) supplemented with 5% fatty acid‐free milk.

For immunoblotting, HeLa cells were solubilized in radioimmunoprecipitation assay (RIPA) buffer (ID#R0278‐50ML, Sigma) supplemented with protease inhibitors (cOmplete ULTRA tablets, #05892970001 Roche) and phosphatase inhibitors (PhosSTOP, #04906845001, Roche). Protein concentration of lysates was determined using a bicinchoninic acid (BCA) assay kit (#23225, Pierce), and equal protein amounts of each sample were migrated on 10 or 12% SDS/polyacrylamide gels. All Western blotting experiments involved the transfer of samples to polyvinylidene difluoride membranes (PVDF, #IPVH00010, MerckMillipore), incubation with primary antibodies or secondary antibodies coupled to horseradish peroxidase, and detection using enhanced chemiluminescence (ECL) Plus reagent (#32132, Pierce). Chemiluminescence was imaged using a ChemiDoc™ MP imaging system (Bio‐Rad).

### 4.6 Infection of human cells

HeLa cells (7 × 10^4^) were seeded in 6‐well plates (Thermo Scientific) and treated with siRNAs as described above. Cell cultures were infected with *S. flexneri* strains at a multiplicity of infection (MOI, bacteria: cell) of 100:1 (*S. flexneri* wildtype and *S. flexneri* Δ*galU*). In the case of *S. flexneri* wildtype, bacteria and cells were immediately centrifuged at 110 × g for 10 min at room temperature. Then, plates were placed at 37 °C and 5% CO_2_ for 30 min. Infected cultures were washed 2× with phosphate‐buffered saline (PBS) pH 7.4 and incubated with fresh DMEM containing 10% FBS and 50 mg/mL gentamicin at 37 °C and 5% CO_2_ for 4 hr.

### 4.7 Confocal microscopy

Hela cells (7 × 10^4^) growing on 22 × 22‐mm glass coverslips were transfected with siRNA for 72 hr (Figures 2c and 3a). After DMSO or STS treatments, cells were fixed in 3% paraformaldehyde (PFA) in PBS for 20 min. Cells were subsequently washed 3X in PBS pH 7.4 and permeabilized 5 min with 0.1% Triton X‐100 in PBS. Cells were then washed 3X in PBS and incubated in blocking buffer (1% bovine serum albumin [BSA] in PBS) for a minimum of 30 min. Cells were probed with primary antibodies diluted in blocking buffer overnight at 4°C. Cells were washed and treated with Alexa Fluor conjugated secondary antibodies for 1 hr, followed by washes and mounting in Prolong Gold anti‐fade reagent (#P36935, Invitrogen). Microscopy images were acquired using a 63x/1.4 C‐Plan Apo oil immersion lens on a Zeiss LSM 880 confocal microscope driven by ZEN Black software (v2.3). Confocal images were processed using Airyscan processing (Weiner filter) using “Auto Filter” and “3D Processing” options.

### 4.8 Zebrafish infections and CRISPR‐Cas9 editing

Animal experiments were performed according to the Animals (Scientific Procedures) Act 1986 and approved by the Home Office (Project license: PPL P4E664E3C). Wildtype zebrafish (AB strain) were used for all experiments. Larvae were reared at 28.5 °C in Petri dishes containing embryo medium, consisting of 0.5x E2 water supplemented with 0.5 ppm (ppm) of methylene blue (Sigma‐Aldrich, St. Louis, Missouri). For injections and in vivo imaging, anesthesia was obtained with buffered 200 μg/mL tricaine (Sigma‐Aldrich) in embryo medium. For CRISPR‐Cas9 editing, three Alt‐R® crisprRNA (crRNA, Integrated DNA technologies) were designed, targeting three consecutive exons of the zebrafish *sept2* gene. For each target, a crRNA/tracrRNA complex was obtained by combining crRNA and Alt‐R® trans‐activating crRNA (tracrRNA) at 60 μM concentration each, in IDTE buffer (10 mM Tris HCl, 0.1 mM EDTA, pH 7.5), following the manufacturer's guidelines. Complexes were aliquoted and stored at −80 °C. 1 μL of Alt‐R® *Streptococcus pyogenes* Cas9 nuclease V3 protein (10 μg/μL, Integrated DNA technologies) was combined with 5 μL of Cas9 buffer (20 mM HEPES; 150 mM KCl, pH 7.5), 0.1 μL of phenol red solution (10%) and 1.5 μL of each of the three crRNA/tracRNA complexes. The solution was incubated at 37 °C for 10 min, returned on ice and microinjected (1 nL/egg) in the yolk sac of zebrafish eggs at a single‐cell stage. As a control, mock injections were performed with an equivalent solution, lacking Cas9 and crRNA/tracrRNA complexes. Efficient targeting was validated by PCR and gel electrophoresis. Sequences of crRNA and PCR primers are reported in Table S2. *S*. *flexneri* M90T or *S. flexneri* Δ*galU* were prepared for injections as in (Torraca et al., 2019). Briefly, bacteria were grown to log phase, spun down, washed in PBS, and resuspended to a density equivalent to an OD_600_ = 18 in an injection buffer containing 2% polyvinylpyrrolidone (Sigma‐Aldrich) and 0.5% phenol red (Sigma‐Aldrich) in PBS. 1 nL (corresponding to ~9,000 CFU) of bacterial suspension was microinjected in the hindbrain ventricle (HBV) of 3 days post‐fertilization (dpf) zebrafish larvae. Bacterial enumeration was performed a posteriori by mechanical disruption of infected larvae in PBS (Sigma‐Aldrich) and plating of serial dilutions onto Congo red‐TSA plates.

### 4.9 Flow cytometry

Following siRNA transfection, HeLa cells were treated with 1 μM staurosporine for 5 hr. Cells were harvested by trypsinization (Sigma, T3924) and collected after stopping the trypsin reaction with 10% (vol/vol) FBS containing medium, followed by washing three times with DPBS (Fisher, 14,190–094), centrifugation (1,500 × g for 5 min), and fixation with 4% PFA for 15 min at RT. After permeabilization with methanol (100%) (Fisher, M/3950/17), overnight at −20 °C, cells were stained (at room temperature for 2 hr) with anti‐cleaved caspase‐3 antibody (diluted 1:500 in dilution buffer (DPBS with 1% (vol/wt) BSA (Sigma, A9647) and 0.3% Triton X‐100. After 2X washing with DPBS, samples were incubated with Alexa Fluor conjugated secondary antibody for 1 hr at room temperature. Cells were analyzed by flow cytometry on a LSRII (BD Biosciences). Data were analyzed with the software FlowJo version 10.7.1.

### 4.10 Quantification and statistics

Image processing and quantifications were performed in Fiji (https://imagej.net/software/fiji/). The Fiji Cell Counter plugin was used (for live apoptosis assays and for fixed cytochrome c or cleaved caspase‐3 assays) by counting the total number of cells in the Hoechst or DAPI channel and the number of cells positive for Annexin V fluorescence, cytosolic cytochrome c staining or cleaved caspase‐3 staining.

Statistical analysis was performed in GraphPad Prism (v8.4, La Jolla, USA). Data are presented as mean ± *SE* of the mean (SEM) from at least three independent experiments per treatment. ANOVA or Student's *t* test were used to compare values, with *p* < .05 considered as significant. Statistical differences for zebrafish survival curves were analyzed using a Log‐rank (Mantel–Cox) test. All statistical details including statistical tests, significance, number of experimental replicates, and cells quantified can be found in the figure legends.

All figures were designed using Adobe Illustrator CC 2018.

## AUTHOR CONTRIBUTIONS

Serge Mostowy conceived and supervised this study.

Hoan Van Ngo, Stevens Robertin, Dominik Brokatzky, Magdalena K. Bielecka, Damián.

Lobato‐Márquez, Vincenzo Torraca, and Serge Mostowy designed the experiments.

Hoan Van Ngo, Stevens Robertin, and Dominik Brokatzky performed experiments using tissue culture cells.

Magdalena K. Bielecka and Vincenzo Torraca performed experiments using zebrafish.

All authors performed data analysis, and took part in the interpretation of results and preparation of materials for the manuscript.

Serge Mostowy wrote the manuscript with comments from all authors.

## CONFLICT OF INTEREST

The authors declare no conflicts of interest.

## Supporting information


**Figure S1** Phylogeny and expression levels of zebrafish septins and Sept2 CRISPR targeting validation. (A) Phylogeny tree of septin proteins from humans (Hsa, *Homo sapiens*), zebrafish (Dre, *Danio rerio*) and yeast (Sce, *Saccharomyces cerevisiae*). Sequences were obtained from the Ensembl database (https://www.ensembl.org/). When multiple splicing isoforms were reported, the Ensembl canonical isoform or the isoform predicted to be the principal isoform was used in the alignment. (B) Expression level of septin genes in zebrafish in reads per kilobase million (RPKM). Data on basal septin expression extracted from RNAseq datasets (Torraca et al., 2019). (C) Gel electrophoresis of PCR products, demonstrating targeting of CRISPR/Cas9 for the three selected targets. S2: *sept2* crispant; C: Control larva.

**Table S1** Zebrafish septins and their corresponding expression value. Expression levels are represented by the mean and standard deviation of the reads per kilobase million (RPKM) from three biological replicates. Data on basal septin expression extracted from RNAseq datasets (Torraca et al., 2019).
**Table S2.** crisprRNA target sequence and PCR primers used in this study.

## Data Availability

The data that support the findings of this study are available from the corresponding author upon request.

## References

[cm21696-bib-0001] Ashida, H. , Suzuki, T. , & Sasakawa, C. (2021). *Shigella* infection and host cell death: A double‐edged sword for the host and pathogen survival. Current Opinion in Microbiology, 59, 1–7. 10.1016/j.mib.2020.07.007 32784063

[cm21696-bib-0002] Ashkenazi, A. , & Dixit, V. M. (1998). Death receptors: Signaling and modulation. Science (New York, N.Y.), 281(5381), 1305–1308. 10.1126/science.281.5381.1305 9721089

[cm21696-bib-0003] Ashkenazi, A. , & Salvesen, G. (2014). Regulated cell death: Signaling and mechanisms. Annual Review of Cell and Developmental Biology, 30, 337–356. 10.1146/annurev-cellbio-100913-013226 25150011

[cm21696-bib-0004] Bock, F. J. , & Tait, S. (2020). Mitochondria as multifaceted regulators of cell death. Nature Reviews Molecular Cell Biology, 21(2), 85–100. 10.1038/s41580-019-0173-8 31636403

[cm21696-bib-0005] Bratton, S. B. , & Salvesen, G. S. (2010). Regulation of the Apaf‐1‐caspase‐9 apoptosome. Journal of Cell Science, 123(Pt 19), 3209–3214. 10.1242/jcs.073643 20844150 PMC2939798

[cm21696-bib-0006] Clark, C. S. , & Maurelli, A. T. (2007). *Shigella flexneri* inhibits staurosporine‐induced apoptosis in epithelial cells. Infection and Immunity, 75(5), 2531–2539. 10.1128/IAI.01866-06 17339354 PMC1865761

[cm21696-bib-0007] Danial, N. N. , & Korsmeyer, S. J. (2004). Cell death: Critical control points. Cell, 116(2), 205–219. 10.1016/s0092-8674(04)00046-7 14744432

[cm21696-bib-0008] Dorstyn, L. , Akey, C. W. , & Kumar, S. (2018). New insights into apoptosome structure and function. Cell Death and Differentiation, 25(7), 1194–1208. 10.1038/s41418-017-0025-z 29765111 PMC6030056

[cm21696-bib-0009] Duggan, G. M. , & Mostowy, S. (2018). Use of zebrafish to study *Shigella* infection. Disease Models & Mechanisms, 11(2), dmm032151. 10.1242/dmm.032151 29590642 PMC5894947

[cm21696-bib-0010] Edison, N. , Curtz, Y. , Paland, N. , Mamriev, D. , Chorubczyk, N. , Haviv‐Reingewertz, T. , … Larisch, S. (2017). Degradation of Bcl‐2 by XIAP and ARTS promotes apoptosis. Cell Reports, 21(2), 442–454. 10.1016/j.celrep.2017.09.052 29020630 PMC5667555

[cm21696-bib-0011] Edison, N. , Zuri, D. , Maniv, I. , Bornstein, B. , Lev, T. , Gottfried, Y. , … Larisch, S. (2012). The IAP‐antagonist ARTS initiates caspase activation upstream of cytochrome C and SMAC/Diablo. Cell Death and Differentiation, 19(2), 356–368. 10.1038/cdd.2011.112 21869827 PMC3263492

[cm21696-bib-0012] Estey, M. P. , Di Ciano‐Oliveira, C. , Froese, C. D. , Bejide, M. T. , & Trimble, W. S. (2010). Distinct roles of septins in cytokinesis: SEPT9 mediates midbody abscission. The Journal of Cell Biology, 191(4), 741–749. 10.1083/jcb.201006031 21059847 PMC2983063

[cm21696-bib-0013] García‐Fernández, M. , Kissel, H. , Brown, S. , Gorenc, T. , Schile, A. J. , Rafii, S. , … Steller, H. (2010). Sept4/ARTS is required for stem cell apoptosis and tumor suppression. Genes & Development, 24(20), 2282–2293. 10.1101/gad.1970110 20952537 PMC2956207

[cm21696-bib-0014] Gomes, M. C. , & Mostowy, S. (2020). The case for modeling human infection in zebrafish. Trends in Microbiology, 28(1), 10–18. 10.1016/j.tim.2019.08.005 31604611

[cm21696-bib-0015] Gottfried, Y. , Rotem, A. , Lotan, R. , Steller, H. , & Larisch, S. (2004). The mitochondrial ARTS protein promotes apoptosis through targeting XIAP. The EMBO Journal, 23(7), 1627–1635. 10.1038/sj.emboj.7600155 15029247 PMC391065

[cm21696-bib-0016] Günther, S. D. , Fritsch, M. , Seeger, J. M. , Schiffmann, L. M. , Snipas, S. J. , Coutelle, M. , … Kashkar, H. (2020). Cytosolic gram‐negative bacteria prevent apoptosis by inhibition of effector caspases through lipopolysaccharide. Nature Microbiology, 5(2), 354–367. 10.1038/s41564-019-0620-5 31873204

[cm21696-bib-0017] Jenner, A. , Peña‐Blanco, A. , Salvador‐Gallego, R. , Ugarte‐Uribe, B. , Zollo, C. , Ganief, T. , … Garcia‐Saez, A. J. (2022). DRP1 interacts directly with BAX to induce its activation and apoptosis. EMBO Journal, 13, e108587. 10.15252/embj.2021108587 PMC9016351 35023587

[cm21696-bib-0018] Kumar, P. , Nagarajan, A. , & Uchil, P. D. (2018). Analysis of cell viability by the MTT assay. Cold Spring Harbor Protocols, 2018(6). 10.1101/pdb.prot095505 29858338

[cm21696-bib-0019] Krokowski, S. , Lobato‐Márquez, D. , Chastanet, A. , Pereira, P. M. , Angelis, D. , Galea, D. , … Mostowy, S. (2018). Septins recognize and entrap dividing bacterial cells for delivery to lysosomes. Cell Host & Microbe, 24(6), 866–874.e4. 10.1016/j.chom.2018.11.005 30543779 PMC6299245

[cm21696-bib-0020] Kroll, F. , Powell, G. T. , Ghosh, M. , Gestri, G. , Antinucci, P. , Hearn, T. J. , … Rihel, J. (2021). A simple and effective F0 knockout method for rapid screening of behaviour and other complex phenotypes. eLife, 10, e59683. 10.7554/eLife.59683 33416493 PMC7793621

[cm21696-bib-0021] Larisch, S. , Yi, Y. , Lotan, R. , Kerner, H. , Eimerl, S. , Tony Parks, W. , … Roberts, A. B. (2000). A novel mitochondrial septin‐like protein, ARTS, mediates apoptosis dependent on its P‐loop motif. Nature Cell Biology, 2(12), 915–921. 10.1038/35046566 11146656

[cm21696-bib-0022] Lembo‐Fazio, L. , Nigro, G. , Noël, G. , Rossi, G. , Chiara, F. , Tsilingiri, K. , … Bernardini, M. L. (2011). Gadd45α activity is the principal effector of *Shigella* mitochondria‐dependent epithelial cell death in vitro and ex vivo. Cell Death & Disease, 2(2), e122. 10.1038/cddis.2011.4 21368893 PMC3101704

[cm21696-bib-0023] Lobato‐Márquez, D. , Krokowski, S. , Sirianni, A. , Larrouy‐Maumus, G. , & Mostowy, S. (2019). A requirement for septins and the autophagy receptor p62 in the proliferation of intracellular Shigella. Cytoskeleton (Hoboken, N.J.), 76(1), 163–172. 10.1002/cm.21453 29752866 PMC6519264

[cm21696-bib-0024] Lobato‐Márquez, D. , Xu, J. , Güler, G. Ö. , Ojiakor, A. , Pilhofer, M. , & Mostowy, S. (2021). Mechanistic insight into bacterial entrapment by septin cage reconstitution. Nature Communications, 12(1), 4511. 10.1038/s41467-021-24721-5 PMC8302635 34301939

[cm21696-bib-0025] Mageswaran, S. K. , Grotjahn, D. A. , Zeng, X. , Barad, B. A. , Medina, M. , Hoang, M. H. , … Jensen, G. J. (2021). Nanoscale details of mitochondrial fission revealed by cryo‐electron tomography. bioRxiv 12.13.472487. 10.1101/2021.12.13.472487

[cm21696-bib-0026] Mamriev, D. , Abbas, R. , Klingler, F. M. , Kagan, J. , Kfir, N. , Donald, A. , … Larisch, S. (2020). A small‐molecule ARTS mimetic promotes apoptosis through degradation of both XIAP and Bcl‐2. Cell Death & Disease, 11(6), 483. 10.1038/s41419-020-2670-2 32587235 PMC7316745

[cm21696-bib-0027] Mazon‐Moya, M. J. , Willis, A. R. , Torraca, V. , Boucontet, L. , Shenoy, A. R. , Colucci‐Guyon, E. , & Mostowy, S. (2017). Septins restrict inflammation and protect zebrafish larvae from *Shigella* infection. PLoS Pathogens, 13(6), e1006467. 10.1371/journal.ppat.1006467 28650995 PMC5507465

[cm21696-bib-0028] Mostowy, S. , & Cossart, P. (2012). Septins: The fourth component of the cytoskeleton. Nature Reviews Molecular Cell Biology, 13(3), 183–194. 10.1038/nrm3284 22314400

[cm21696-bib-0029] Mostowy, S. , & Shenoy, A. R. (2015). The cytoskeleton in cell‐autonomous immunity: Structural determinants of host defence. Nature Reviews Immunology, 15(9), 559–573. 10.1038/nri3877 PMC4869833 26292640

[cm21696-bib-0030] Mostowy, S. , Bonazzi, M. , Hamon, M. A. , Tham, T. N. , Mallet, A. , Lelek, M. , … Cossart, P. (2010). Entrapment of intracytosolic bacteria by septin cage‐like structures. Cell Host & Microbe, 8(5), 433–444. 10.1016/j.chom.2010.10.009 21075354

[cm21696-bib-0031] Mostowy, S. , Boucontet, L. , Mazon Moya, M. J. , Sirianni, A. , Boudinot, P. , Hollinshead, M. , … Colucci‐Guyon, E. (2013). The zebrafish as a new model for the in vivo study of *Shigella flexneri* interaction with phagocytes and bacterial autophagy. PLoS Pathogens, 9(9), e1003588. 10.1371/journal.ppat.1003588 24039575 PMC3764221

[cm21696-bib-0032] Naderer, T. , & Fulcher, M. C. (2018). Targeting apoptosis pathways in infections. Journal of Leukocyte Biology, 103(2), 275–285. 10.1189/JLB.4MR0717-286R 29372933

[cm21696-bib-0033] Pagliuso, A. , Tham, T. N. , Stevens, J. K. , Lagache, T. , Persson, R. , Salles, A. , … Stavru, F. (2016). A role for septin 2 in Drp1‐mediated mitochondrial fission. EMBO Reports, 17(6), 858–873. 10.15252/embr.201541612 27215606 PMC5278612

[cm21696-bib-0034] Robertin, S. , & Mostowy, S. (2020). The history of septin biology and bacterial infection. Cellular Microbiology, 22(4), e13173. 10.1111/cmi.13173 32185906

[cm21696-bib-0035] Salvesen, G. S. , & Ashkenazi, A. (2011). Snapshot: Caspases. Cell, 147(2), 476–476.e1. 10.1016/j.cell.2011.09.030 22000022

[cm21696-bib-0036] Sirianni, A. , Krokowski, S. , Lobato‐Márquez, D. , Buranyi, S. , Pfanzelter, J. , Galea, D. , … Mostowy, S. (2016). Mitochondria mediate septin cage assembly to promote autophagy of *Shigella* . EMBO Reports, 17(7), 1029–1043. 10.15252/embr.201541832 27259462 PMC4931556

[cm21696-bib-0037] Spiliotis, E. T. , & Nakos, K. (2021). Cellular functions of actin‐ and microtubule‐associated septins. Current Biology: CB, 31(10), R651–R666. 10.1016/j.cub.2021.03.064 34033796 PMC8194058

[cm21696-bib-0038] Thornberry, N. A. , & Lazebnik, Y. (1998). Caspases: Enemies within. Science (New York, N.Y.), 281(5381), 1312–1316. 10.1126/science.281.5381.1312 9721091

[cm21696-bib-0039] Torraca, V. , & Mostowy, S. (2018). Zebrafish infection: From pathogenesis to cell biology. Trends in Cell Biology, 28(2), 143–156. 10.1016/j.tcb.2017.10.002 29173800 PMC5777827

[cm21696-bib-0040] Torraca, V. , Kaforou, M. , Watson, J. , Duggan, G. M. , Guerrero‐Gutierrez, H. , Krokowski, S. , … Mostowy, S. (2019). *Shigella sonnei* infection of zebrafish reveals that O‐antigen mediates neutrophil tolerance and dysentery incidence. PLoS Pathogens, 15(12), e1008006. 10.1371/journal.ppat.1008006 31830135 PMC6980646

[cm21696-bib-0041] Van Ngo, H. , & Mostowy, S. (2019). Role of septins in microbial infection. Journal of Cell Science, 132(9), jcs226266. 10.1242/jcs.226266 31040222

[cm21696-bib-0042] Woods, B. L. , & Gladfelter, A. S. (2021). The state of the septin cytoskeleton from assembly to function. Current Opinion in Cell Biology, 68, 105–112. 10.1016/j.ceb.2020.10.007 33188984 PMC7952027

