# Supplementary figures and images for: Septins promote caspase activity and coordinate mitochondrial apoptosis
Source: Cytoskeleton (Hoboken). 2022 May 9;80(7-8):254–65. doi: 10.1002/cm.21696 (PMC10952901; doi:10.1002/cm.21696)

# SUPPLEMENTARY FIGURE 1

**A**

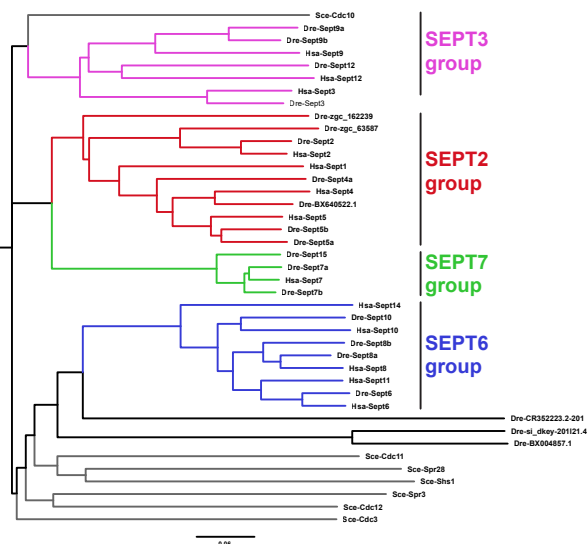

**B**

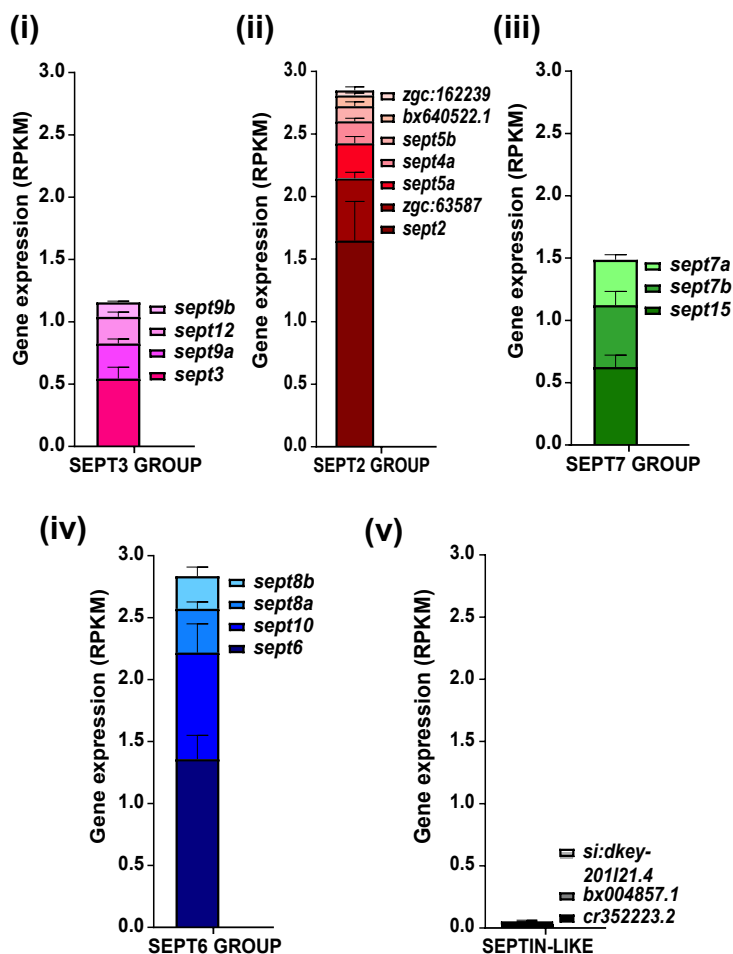

**C**

## Target Sept2.1AA

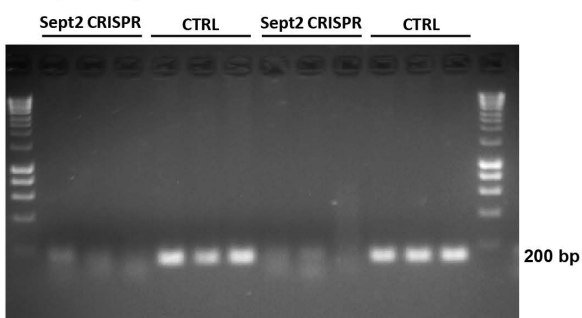

## Target Sept2.1AB

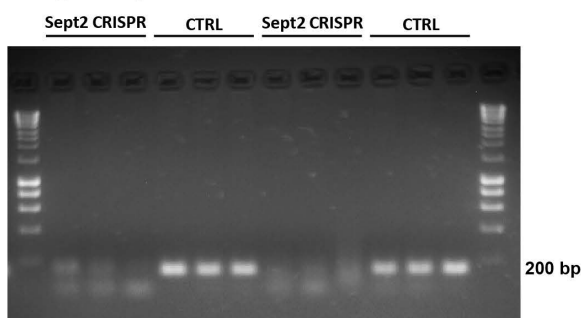

## Target Sept2.1AC

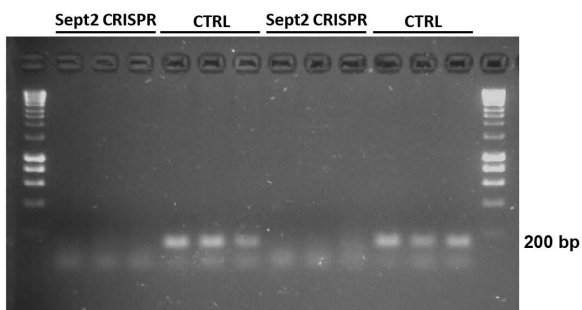

Supplement: Supplementary file 1 — Figure S1 Phylogeny and expression levels of zebrafish septins and Sept2 CRISPR targeting validation. (A) Phylogeny tree of septin proteins from humans (Hsa, Homo sapiens), zebrafish (Dre, Danio rerio) and yeast (Sce, Saccharomyces cerevisiae). Sequences were obtained from the Ensembl database (https://www.ensembl.org/). When multiple splicing isoforms were reported, the Ensembl canonical isoform or the isoform predicted to be the principal isoform was used in the alignment. (B) Expression level of septin genes in zebrafish in reads per kilobase million (RPKM). Data on basal septin expression extracted from RNAseq datasets (Torraca et al., 2019). (C) Gel electrophoresis of PCR products, demonstrating targeting of CRISPR/Cas9 for the three selected targets. S2: sept2 crispant; C: Control larva. [file CM-80-254-s002.pdf]
