# Supplementary material for: Septins promote caspase activity and coordinate mitochondrial apoptosis
Source: Cytoskeleton (Hoboken). 2022 May 9;80(7-8):254–65. doi: 10.1002/cm.21696 (PMC10952901; doi:10.1002/cm.21696)
Supplement: Supplementary file 2 — Table S1 Zebrafish septins and their corresponding expression value. Expression levels are represented by the mean and standard deviation of the reads per kilobase million (RPKM) from three biological replicates. Data on basal septin expression extracted from RNAseq datasets (Torraca et al., 2019). Table S2. crisprRNA target sequence and PCR primers used in this study. [file CM-80-254-s001.docx]

**Table S1.** Zebrafish septins and their corresponding expression value. Expression levels are represented by the mean and standard deviation of the reads per kilobase million (RPKM) from three biological replicates. Data on basal septin expression extracted from RNAseq datasets in (Torraca et al.*,* 2019).

| **SEPTIN GROUP** | **Septin gene name** | **Reads per Kilobase million** | |
| --- | --- | --- | --- |
|  |  | **Mean** | **St. deviation** |
| SEPT3 GROUP | *sept3* | 0.54 | 0.09 |
|  | *sept9a* | 0.28 | 0.04 |
|  | *sept12* | 0.21 | 0.04 |
|  | *sept9b* | 0.12 | 0.01 |
| SEPT2 GROUP | *sept2* | 1.65 | 0.31 |
|  | *zgc:63587* | 0.50 | 0.05 |
|  | *sept5a* | 0.28 | 0.05 |
|  | *sept4a* | 0.18 | 0.02 |
|  | *sept5b* | 0.12 | 0.03 |
|  | *bx640522.1* | 0.09 | 0.02 |
|  | *zgc:162239* | 0.04 | 0.03 |
| SEPT7 GROUP | *sept15* | 0.63 | 0.09 |
|  | *sept7b* | 0.50 | 0.11 |
|  | *sept7a* | 0.36 | 0.04 |
| SEPT6 GROUP | *sept6* | 1.36 | 0.19 |
|  | *sept10* | 0.86 | 0.23 |
|  | *sept8a* | 0.35 | 0.05 |
|  | *sept8b* | 0.26 | 0.07 |
| SEPTIN LIKE | *cr352223.2* | 0.03 | 0.00 |
|  | *bx004857.1* | 0.01 | 0.01 |
|  | *si:dkey-201l21.4* | 0.01 | 0.01 |

**Table S2.** crisprRNA target sequence and PCR primers used in this study.

| **Target name** | **Target sequence** | **Forward genotyping primer** | **Reverse genotyping primer** | **Genotyping product size (in WT)** |
| --- | --- | --- | --- | --- |
| Sep2.1 AA | TGGTCGACACACCTGGATACGGG | AGATTGAGCGTACTGTGCAGAT | AATGATCATAGCAATCCTCCTCAC | 145 bp |
| Sept2.1AB | TTTGCGAACCCAACATACCCAGG | TTACCGACCACCATGAGAGTG | CATGCTCATAGATCTGTTCTCTCTT | 150 bp |
| Sept2.1AC | GCACCAGGAATGACCCTCTCTGG | TCAGGCCCGACATTTACAAAAACA | AGTCTGGTTTGGGGAAGTCAAC | 149 bp |
